# Scaling‐up school mental health services in low resource public schools of rural Pakistan: the Theory of Change (ToC) approach

**DOI:** 10.1186/s13033-021-00435-5

**Published:** 2021-01-12

**Authors:** Syed Usman Hamdani, Zill-e- Huma, Nadia Suleman, Azza Warraitch, Naila Muzzafar, Midhat Farzeen, Fareed Aslam Minhas, Atif Rahman, Lawrence S. Wissow

**Affiliations:** 1 University of Liverpool, Liverpool, UK; 2 Human Development Research Foundation, Islamabad, Pakistan; 3 Institute of Psychiatry, Rawalpindi, Pakistan; 4 University of Washington, Seattle, USA

**Keywords:** School mental health, Public schools, Theory of Change, Scale-up, Child mental health, Low resource settings

## Abstract

**Background:**

Ninety percent of children with mental health problems live in low or middle-income countries (LMICs). School-based programs offer opportunities for early identification and intervention, however implementation requires cross-sector collaboration to assure sustainable delivery of quality training, ongoing supervision, and outcomes monitoring at scale. In Pakistan, 35% of school-aged children are reported to have emotional and behavioral problems. As in many other LMICs, the government agencies who must work together to mount school-based programs have limited resources and a limited history of collaboration. The “Theory of Change” (ToC) process offers a way for new partners to efficiently develop mutual goals and long-term prospects for sustainable collaboration.

**Objective:**

Develop a model for scale-up of school based mental health services in public schools of Pakistan.

**Methods:**

We used ToC workshops to develop an empirically supported, ‘hypothesized pathway’ for the implementation of WHO’s School Mental Health Program in the public schools of rural Pakistan. Three workshops included 90 stakeholders such as policy makers from education and health departments, mental health specialists, researchers, head teachers, teachers and other community stakeholders including non-governmental organizations.

**Results:**

The ToC process linked implementers, organizations, providers and consumers of school mental health services to develop common goals and relate them (improved child socioemotional wellbeing, grades and participation in activities) to interventions (training, monitoring and supervision of teachers; collaboration with parents, teachers and primary health care facilities and schools). Key testable assumptions developed in the process included buy-in from health care providers, education officials and professionals, community-based organizations and families. For example, teachers needed skills for managing children’s problems, but their motivation might come from seeking improved school performance and working conditions. Poverty, stigma and lack of child mental health literacy among teachers, administration, and parents were identified as key hypothesized barriers. Children and their families were identified as key stakeholders to make such a program successful.

**Discussion:**

ToC workshops assisted in team building and served as a stakeholders’ engagement tool. They helped to develop and support testable hypotheses about the structures, collaborations, and knowledge most important to scaling-up school based mental health services in Pakistan.

## Background

Child and adolescent mental health has been identified as a potential entry point for early interventions across the life-course to prevent the burden of mental health problems in later life [1]. Schools offer an opportunity for early intervention to promote child and adolescent mental health by improving social, behavioral, emotional and academic functioning of children and adolescents [2–5]. Schools also provide opportunities for cross-sectoral collaborations that help build resilient societies [6]. Following consultations with international and regional experts and stakeholders, the World Health Organization’s Eastern Mediterranean Region Office (WHO EMRO) developed a School Mental Health Program (SMHP) endorsed by member countries, including Pakistan. The SMHP aims to empower educators to understand the importance of mental health in schools, enhance educators’ understanding of child development, and allow them to identify and support children at-risk of socio-emotional problems.

Pakistan is among the low and middle income countries of South Asia with an approximate population of 200 million [7]. The reported prevalence rates of child and adolescent’s mental health problems in Pakistan are much higher (35%) than the global prevalence estimates of 10–20% [8, 9]. To address the burden of child and adolescent mental health problems in Pakistan, the Ministry of National Health Services adopted the SMHP under the WHO EMRO regional framework [10] for mental health. Implementation will take place in a pilot district to test assumptions and develop a scalable and sustainable pathway for school based mental health programs across Pakistan.

Like many other public health interventions, the SMHP involves multiple components that are expected to be delivered at multiple levels by different stakeholders. This complexity makes it difficult to implement the program, especially in low resource school settings in Pakistan. Thus, to pilot the SMHP, the Ministry of National Health Services supported the development of SHINE, the School Health Implementation Network, Eastern Mediterranean region.^1^ SHINE is one of the collaborative mental health research hubs funded by the US National Institute of Mental Health. Along with its partner hubs, SHINE is devoted to better understanding how child mental health interventions can be brought to scale in low and middle-income countries. The SHINE hub is particularly focused on the challenges of implementing school mental health programs, and includes partners in several other WHO EMRO member countries.

Recently various theory-driven approaches have been used to understand how and why complex programs work [11, 12]. SHINE used the Theory of Change (ToC) approach [13] to plan the scale-up of School Mental Health Program in Pakistan. ToC is distinct from other approaches as it not only focuses on how to create social change by empowering individuals, but it also makes explicit the causal pathways through which change may happen [14, 15]. The output of ToC workshops, the ToC map, is a theory-supported hypothetical visual pathway which demonstrates how a public health intervention can bring about specific long-term change through a logical sequence of intermediate outcomes [16–18]. This paper describes the process and outcomes of using the ToC approach to involve stakeholders to identify gaps in the implementation of the SMHP, and how these gaps could be addressed by developing contextually relevant interventions and harnessing multi-sectoral partnerships to deliver the SMHP at-scale in the public schools of rural Rawalpindi, Pakistan.

## Methods

### Settings

#### Implementation partners and stakeholders for the SMHP

Although the implementation of the SMHP is taking place under the leadership of Ministry of National Health Services, multi-sectoral collaboration among national, provincial, district and local authorities from education and health departments was considered crucial. SHINE’s primary partner in Pakistan, the Institute of Psychiatry-World Health Organization Collaborating Centre for mental health research and training (IoP-WCC) in Rawalpindi, had been assigned by the Ministry of National Health Services to lead the implementation of the SMHP in a pilot district in partnership with the District Education Authority, Rawalpindi. School education is the responsibility of the Ministry of Education in each province of Pakistan. District education departments, within each provincial education department, are the administrative hubs for educational activities in respective districts. Particularly relevant to the SMHP’s training goals, it is the district departments’ responsibility to organize Continuous Professional Development courses/workshops for public school teachers.

Three ToC workshops with 90 stakeholders from education and health departments were conducted over a 12-month period. In Pakistan, the SMHP is being implemented by the Ministry of Health and the WHO, in collaboration with the academic and implementation partners. The participants for ToC workshops were identified by the research team from the existing networks of these organizations. The composition of the stakeholders was decided depending upon the relevance of the stakeholders to the program. Stakeholders represented policy makers, representatives from district education planners and officers, mental health specialists, researchers, head teachers and teachers. The description of stakeholders is given in Table 1.

**Table 1:** Stakeholders of ToC workshops

Participants of ToC workshops (N = 90)	*F*(%)
International partners (Representatives from WHO country and regional offices, international child and adolescent mental health experts and iNGO working on child and adolescent mental health)	8
National partners (Representatives of Ministry of National Health Services, NGOs, private practitioners, public health experts, psychologists, psychiatrists, representatives of parent associations)	12
Representatives from education department (District Education Officers, Assistant Education Officers)	16
Representatives from health department (District Health Officer, Primary Health Care staff)	17
Representatives from IoP-WCC (WHO SMHP implementation team)	23
Head teachers and teachers from public schools in pilot district	14

#### Theory of change (ToC) workshops

The ToC workshops were conducted at the IoP-WCC in Rawalpindi. The workshops were conducted iteratively early on in the planning process to a) to identify key steps in the SMHP implementation pathway, b) to understand the complex interaction of contextual factors that could impact implementation, and c) map out the chain of interdependent pre-conditions necessary for the delivery of school mental health program in public schools of rural Pakistan. The overall goal of the workshops was to develop testable hypotheses, that, after further information gathering, would lead to developing a final implementation plan for the SMHP.

We used a participatory approach to bring together a range of stakeholders to develop a ToC road-map and to encourage stakeholders’ buy-in. Each of the ToC workshop was conducted to achieve specific objectives. The 1st and 2nd ToC workshops were conducted with international and national stakeholders, representatives from the Ministry of Health, WHO, district education and health departments. The objectives of the 1st ToC workshop were to a) orient stakeholders about the WHO SMHP, b) obtain stakeholders’ buy-in and c) identify a mutually agreeable ultimate outcome for the SMHP in Pakistan. A rapid needs assessment for school based mental health services in Pakistan was then conducted based on recommendations from the stakeholders from the 1st ToC workshop.

Informed by the results of the needs assessment (*the findings are reported elsewhere*), the stakeholders in the 2nd workshop identified specific intervention strategies as well as potential challenges and ways to address them. The 3rd ToC workshop was conducted with personnel from district education departments (school heads and teachers) to operationalize the intervention strategies using existing resources and opportunities in schools. The facilitators presented the SMHP as it might have been delivered to teachers using a lecture and role play format; this generated suggestions for changes to both content and method of delivery. The feedback from stakeholders was collected and incorporated in the school mental health program intervention and implementation plans.

### Procedure

A key goal of the ToC workshops was to elicit stakeholders’ buy-in by intimately involving them in the development of the implementation plan for the SMHP. The ToC workshops were led by a trained public mental health expert (UH), based at the IoP-WCC, assisted by trained facilitators. Unlike other stakeholders’ consultation meetings [14] such as focus group discussions or in-depth interviews, which use semi-structured interview guides to elicit information, the major role of the facilitators was to orient the participants about the school mental health initiative and engage them in designing an implementation plan. To start with, the current state of the child mental health services and content of the SMHP was written on the left side of the whiteboard and agreeable ultimate outcomes of the implementation of SMHP, identified by the stakeholders, were written on right-side of the whiteboard. The participants were encouraged to develop a hypothesized pathway and state the required pre-conditions. Subsequently and throughout the workshops, the facilitators posed questions to stakeholders to generate discussions around interventions, short, intermediate, and long-term outcomes, indicators, and assumptions for the program implementation. Brainstorming sessions were conducted in small groups. The discussions were audio-recorded and transcribed by the research team.

### Data analysis

An inductive approach of thematic analysis was used to generate themes from the collected data [19]. At first, we familiarized ourselves by reading and re-reading the transcribed data followed by manual coding, keeping in view the research question and components of ToC map (e.g. short, intermediate and longer outcomes, pre-conditions, indicators and assumptions). The relevant data associated with each code was clustered together to form themes. Themes were revised and finalized in relation to the data and were given names. The themes were then placed under each component of ToC map. An initial ToC road-map for the SMHP was developed after the first workshop and modified after each of the following workshops. The findings of each ToC workshop were presented to the stakeholders in next consultative workshop to refine the ToC map and identify additional resources and interventions required to complete each pre-conditions. Finally, a consolidated roadmap containing assumptions, outcomes, pre-conditions, implementation interventions and indicators for the implementation of school mental health program was developed.

### Ethics approval

The ethics approval to conduct the ToC workshops were obtained from ethics review committees of University of Liverpool (UoL), UK and the Human Development Research Foundation (HDRF), Pakistan.

## Results

**Fig. 1:**
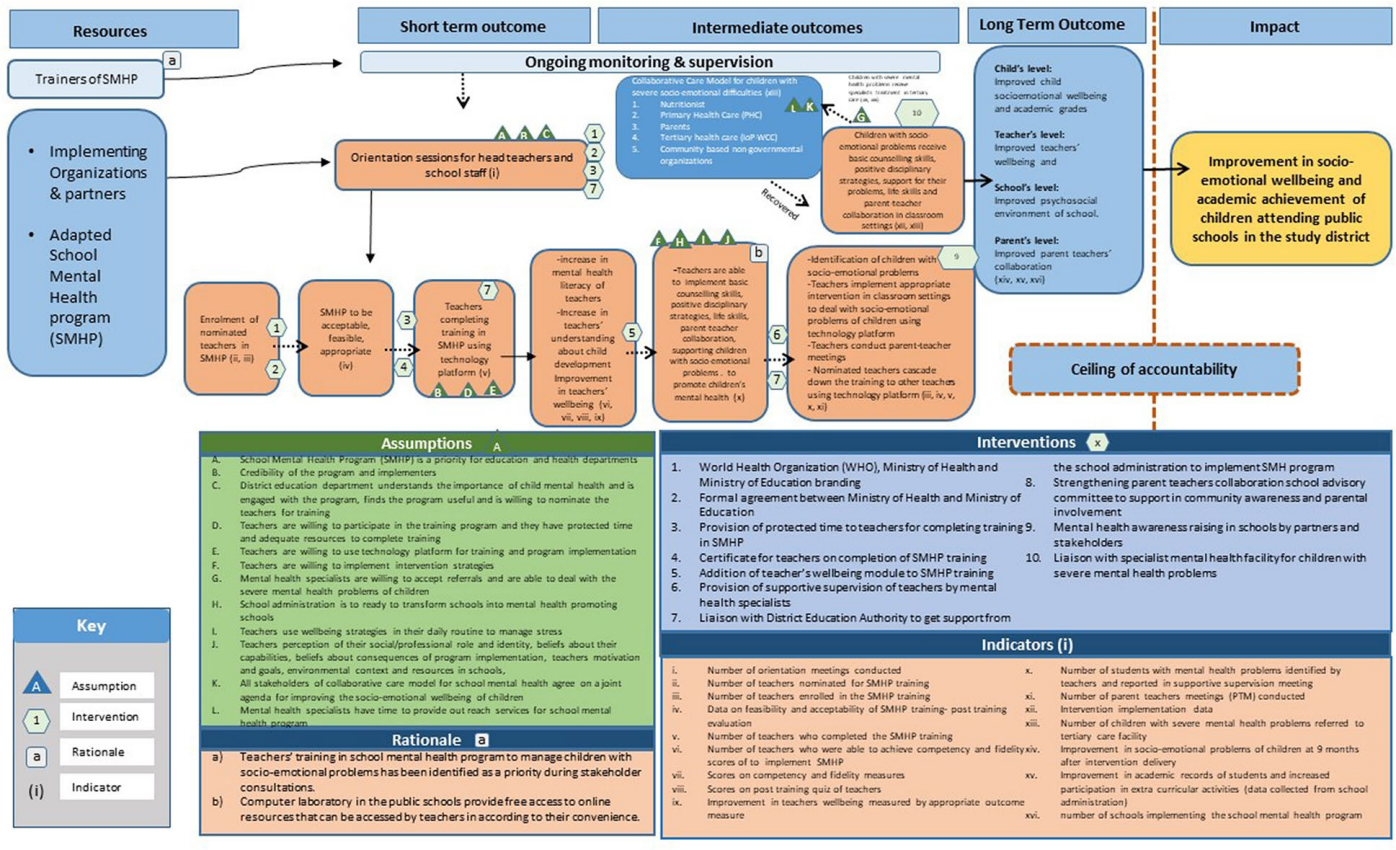
Theory of Change (ToC) map for the implementation of School Mental Health Program (SMHP) in public schools of rural Pakistan

The ToC approach successfully served as a ‘planning and team building tool’ for the SMHP. In addition to securing the commitment and collaboration of stakeholders across disciplines and government levels, the workshops were instrumental in understanding the outcomes that the stakeholders collectively supported and valued; the context in which the SMHP would be implemented; methods that might or might not be feasible; and the resources that could be leveraged or that would have to be supplied. Short-term, intermediate and long-term goals for the program were identified and operationalized in terms of program indicators and outcomes. Key assumptions and pre-conditions for each outcome and possible indicators to measure each outcome were identified and operationalized on the ToC map (Fig. 1).

### The context

The socioecological context in which the SMHP is being implemented emerged as a significant factor to influence implementation.

1. Lack of dedicated human resources: Implementing the SMHP with fidelity and at-scale faced the challenge of a lack of dedicated staff such as school counselors or psychologists to ensure quality training, supervision and monitoring. In addition, a principle concern of the stakeholders was around allocation of teachers’ time and effort in training and delivery of SMHP interventions to students. As the public school system is struggling to meet educational demands, addition of the SMHP could overburden the teachers and could potentially compromise the educational outcomes of the schools.
2. Impoverished school environment: The stakeholders identified lack of financial, human and material resources such as overcrowded classrooms, low number of teachers, a high student to teacher ratio and inadequate infrastructure needed for optimal delivery of education services in rural schools of Pakistan.
3. Lack of parental cooperation: A frequent concern of stakeholders was lack of cooperation and involvement of parents in their children’s educational activities. Most residents of rural communities in Pakistan are illiterate. In addition, most are dependent on subsistence agricultural and farming activities for their living and thus many suffer from financial and domestic problems. The stakeholders felt that parents’ low level of literacy and high levels of poverty posed barriers to engagement with schools.
4. Stigma related to mental health problems: Lack of mental health awareness and stigma among teachers, school administration and parents was identified as a key barrier to achieve the long-term outcomes of school mental health program.

### Outcomes for school mental health program identified by stakeholders

The stakeholders identified improved socio-emotional wellbeing and academic performance of school going children; improved wellbeing of teachers and improved psychosocial environment of school in the implementation district as the long-term outcomes of school mental health program. Teachers’ program implementation behavior was identified as the key intermediate program outcome. Program implementation behavior was defined as teachers’ belief about their capabilities, their motivation, willingness to get trained and then implement the school mental health program in schools, environmental context and resources and social influences were thought to be the potential determinants of teachers’ behavior change. Nomination of teachers for training in the SMHP by the district education department, improved mental health literacy and wellbeing of teachers as a result of training were identified as the short-term outcomes of school mental health program implementation.

The stakeholders developed a causal pathway linking long-term, intermediate and short-term outcomes that are critical to achieve the ultimate outcomes. The stakeholders hypothesized that the training of teachers will improve teachers’ program implementation behavior, which in turn will lead to improved teacher competency and fidelity, self-efficacy and wellbeing and will result in improved socio-emotional wellbeing of children, in addition to enhancing the school environment and parental involvement.

### Indicators identified by stakeholders

The stakeholders identified indicators for each program outcome. These indicators include; number of schools implementing the school mental health program; number of school teachers trained and implementing the group intervention sessions to children; competency and intervention fidelity data of school teachers; change in teachers program implementation behavior; number of children with socio-emotional problems that are identified and referred to specialist mental health facility and improvement in socio-emotional wellbeing of children measured using psychometrically strong questionnaire. The stakeholders identified lack of human resources, logistical and financial challenges associated with gathering data related to program implementation.

### Underlying assumptions highlighted by the stakeholders

Stakeholders identified key assumptions for the scale-up of the SMHP. Among many competing demands, child mental health might not be considered a priority by health and education departments; school administration or teachers might find it difficult to participate in the program due to excessive workload. Head teachers’ buy-in, flexible timings for teachers’ training, and provision of access to the training course for teachers were other key assumptions highlighted by the stakeholders. Stigma related to mental health problems can be a barrier to the acceptance of the program by the community stakeholders including parents and teachers. The specialists at tertiary mental health facilities might find it difficult to provide services to children with mental health problems due to excessive workload.

### Pre-conditions identified by stakeholders

The stakeholders identified a number of preconditions for the implementation of the SMHP. These included buy-in from the education department, parents and community members.

### Challenges identified in the delivery of school mental health program

A number of implementation challenges were identified by the stakeholders in the implementation of the school mental health program at-scale. These included sustainable delivery of quality training and supervision at-scale; routine monitoring of outcomes and mechanisms for ensuring program quality.

## Recommendations by stakeholders

A number of ‘system and program level interventions’ were suggested by the stakeholders to achieve the goals of school mental health program.

## System level interventions

The system level interventions included;

### Interventions to increase collaboration between multi-stakeholders

Collaboration between multi-stakeholders and buy-in from community is crucial for the implementation of school mental health program in public schools of a rural sub-district in Pakistan. This can be achieved by;

- Formalizing an agreement between health and education departments;
- Increasing mental health awareness in the community and among school administration, teachers and parents by organizing mental health awareness seminars.
- Strengthening school advisory committees to create awareness about significance of school based mental health programs and increase parental involvement. Stakeholders suggested that ensuring involvement of parents with low socio-economic status and improving the infrastructure in public schools will require solutions beyond the health and education departments such as education authorities to reach out to social safety nets and mobilizing community resources to meet the financial needs of public schools and families in rural communities.
- The need to establish a collaborative care model for school mental health, consisting of mental health specialists, primary health care staff, school nutritionists, school administration, community based non-governmental mental health organization and parents to cater for the mental health needs of children was considered vital for successful implementation of program and.
- Engaging mental health specialists, primary health care staff, school nutritionists, school administration, community based non-governmental mental health organization and parents through consultative workshops were considered crucial to increase mental health awareness and stakeholders’ buy-in.
- Adaptation of the SMHP to fit the local context as it was originally developed by WHO EMRO;
- Organizing orientation sessions on the SMHP for school staff and parents;
- Use of technology platforms for training teachers and collecting program monitoring data sustainably and at-scale.

### Interventions to increase teachers’ motivation to deliver school mental health program

The stakeholders recommended a number of strategies to increase teachers buy-in into the training. These included;

- The stakeholders identified motivated teachers as a potential human resource in each school who could be trained in the intervention. Each trained teacher can be responsible for cascading training within their own school setting, providing the primary point of contact for day-to-day supervision of other teachers in their schools. Trained teachers would be supported by their peer network in the school, which is in turn will be supervised by mental health experts based at the WHO collaborating center at the Institute of Psychiatry. Over time, such trained teachers could become a resource for further intervention scale-up through their experiences, also acting as ‘champions of school mental health program’ and agents of change within the schools, community, and education system. Apart from the teaching staff, administrative staff such as Assistant Education Officers (AEOs) were suggested to be critical for implementation and quality assurance of program delivery in Pakistan. An AEO is a mobile administrator and responsible for the at-least 15-22 schools of his/her area (*markez)*. He/she supervises the performance of these schools and does the routine monitoring and supervision and management of the personnel in the schools. Involvement of school heads, administrative staff and parents were identified as critical components of program delivery.
- Organizing training of teachers in SMHP in schools. For the program to be effective; teachers should be able to access the training within the school premises, receive support in the implementation of intervention strategies in classroom settings; conduct group intervention sessions with children and receive on-going support from mental health professionals for children as well their own wellbeing. Teachers should be able to identify children with socio-emotional problems; children with severe mental health problem or self-harm and suicidal ideations should be referred to specialist mental health facilities where needed.
- Prioritizing teachers’ wellbeing as integral part of school mental health initiatives;
- Awarding ‘certificate of recognition’ to teachers on completing training course; and.

Other strategies included making school mental health training as a part of teachers’ induction or in-service training. These system level interventions were thought to be critical for implementation and uptake of the program in education department.

## Program level content and delivery adaptations

A number of adaptations were suggested by the stakeholders to the content and delivery of the SMHP to make it suitable for implementation in public schools of rural Rawalpindi.

### Content adaptations to school mental health program

i. Teachers’ wellness: Stakeholders identified teacher’s wellness as a key prerequisite for program implementation by the teachers. To promote teacher’s well-being, self-efficacy and motivation, a new module on teachers’ wellness has been added to provide teachers with self-care strategies. The intervention strategies are adapted from an evidence-based curriculum, developed by the WHO for managing problems in everyday life (20).
ii. The introduction of a collaborative care model with focus on parent-teacher collaboration (21); The stakeholders identified challenges faced by teachers in engaging parents/primary caregivers, especially living in poorer households and difficulties in operationalizing the parental intervention strategies suggested in the school mental health program. To address these challenges, a collaborative care model with school teachers, parents/primary caregivers and primary health care providers as key components has been added to the intervention.
iii. Operationalization of intervention strategies: An important critique of the WHO school mental health program by the stakeholders was that while school mental health program instructs teachers on what to do, it does not explain in detail how to do it in real world settings. To address this challenge, a number of intervention strategies have been re-organized and operationalized for standardized implementation in typical low-income classroom settings (see Table 2 for the description of key components of school mental health program). For example, a structured guideline on how to conduct circle time activities in schools has been developed in consultation with stakeholders and has been incorporated in the intervention.

**Table 2:** Summary of adaptations to school mental health program

Components of school mental health program	Conventional	Suggested adaptations
Knowledge about child development	Theoretical descriptions of each developmental stage through lectures	Use of pictures and narratives through electronic medium to demonstrate different stages and domains of child development
Universal strategies		
Counselling skills	Good communication skills listed in written materials, with limited practice through role play during training	Good communication skills to be integrated throughout the entire training; teachers to be provided examples of good practices through electronic medium
Role of parents/primary caregivers	Brief narrative to explain the role of parents/primary caregivers in child’s education covered in lecture	A collaborative care model to promote parent-teacher collaboration to be added. Detailed guidelines to conduct effective parent teacher meetings to be provided as supplementary material
Promoting life skills–use of circle time	A list of potential life skills which can be implemented in school settings is provided. A brief narrative on what is circle time activity in school settings is provided	Detailed guidelines for teachers on how to impart life skills training in school settings to be incorporated in circle time activities Structured guidelines on how to conduct circle time activities in schools to be developed and incorporated in the intervention
Teachers’ wellness	Brief description in manual and reference to additional reading material as supplementary	A new module to promote teacher`s wellness incorporating evidence-based strategies for stress management to be developed
Targeted strategies	Case studies on anxiety, separation anxiety/school refusal, post-trauma, depression, suicide, ADHD, autism, psychosis, conduct problems, substance use problems to demonstrate what strategy a teacher can implement in classroom settings	A trans-diagnostic approach was recommended to avoid diagnostic labels and to prevent labelling/stigmatization of children in school settings To facilitate teacher’s understanding of the conditions depression, anxiety and PTSD case studies to be combined as internalizing problems case study. Case studies on ADHD and conduct problems to be combined as externalizing problems case study A case study on child with learning difficulties has to be added Case studies not to just demonstrate what to do but also to emphasize how to implement intervention strategies in a classroom setting
Training	A one-off training consisting of didactic lectures to a few nominated teachers on designated dates and venues The content is delivered in the form of didactic lectures	A self-paced, open access, online teachers training course, comprising of interactive videos and role plays to be developed and delivered using a cascade model of training whereby champion teachers cascade down training to all teachers in their respective schools
Supervision	Monthly face to face supervision	A cascade model of monthly face to face supervision meetings whereby trainers supervise the champion teachers who will supervise the school teachers in their respective schools
iv. Addition of intervention strategies for children with learning difficulties: The consultative meetings with the stakeholders identified the need for intervention strategies for managing children with learning difficulties. To cater for this need, a section of intervention strategies for managing a child with learning difficulties in classroom settings have been included in school mental health program
v. Trans-diagnostic approach: While school mental health program is a category-based intervention, the stakeholders identified the need to focus on common emotional and behavioral problems faced by teachers within the classroom settings rather than specific mental health conditions of children

### Delivery adaptations to school mental health program

The stakeholders identified a need to develop sustainable mechanisms for delivery of quality training and supervision to teachers as a part of school mental health program implementation and collection of impact data. The stakeholders recommended development of a technology platform for training of nominated teachers at-scale with fidelity in the intervention strategies and collection of program impact data.

## Discussion

The Theory of Change (ToC) workshops provided us with an opportunity to explore schools as new grounds to improve the socio-emotional well-being of children in low resource rural settings of Pakistan. ToC workshops served as a critical team-building and engagement tool that allowed partnerships to be developed between multidisciplinary team of stakeholders to identify a common goal and work collaboratively to achieve it. The participatory process of the ToC approach, engaging multisector and multilevel stakeholders, led to the development of a mutually agreeable, hypothesized implementation pathway to scale-up school mental health program in public schools of rural Rawalpindi, Pakistan. The stepwise approach to the ToC development allowed stakeholders to discuss in detail the hypothesized outcomes required along the causal pathway. Novel additions to the SMHP that came out of the process included the need of developing a collaborative care model for school based mental health programs and a focus on teachers’ wellbeing and working conditions as key motivating factors for school staff to participate in training. To promote sustainability and integration of the SMHP within the education sector, training can be accredited as pre-service or in-service training course for school teachers.

While schools are an attractive setting to promote mental health in children and adolescents, there is limited evidence on the effectiveness of universal school mental health program at-scale from low and middle income countries (6, 22). This calls for careful attention to the context of delivery of such intervention programs in low resource settings. We used the Theory of Change (ToC) approach to inform the scaled-up implementation of school mental health program in Pakistan which highlighted key contextual factors such as poverty, stigma related to mental illness, lack of resources, and impoverished school environment, which could impede the benefits of such a program at-scale. Stakeholders identified key pre-conditions that could underpin the successful delivery of school mental health program in Pakistan such as addressing competing demands on teachers and school staff’s time and making child mental health a priority by health and education departments.

Although the WHO school mental health program is an evidence informed intervention package, a number of significant adaptations were suggested by the stakeholders in the ToC workshops to the content and delivery of the program to promote adoption, sustainability and scale-up of program in public schools of rural Pakistan. This is supported by the recent literature from implementation science research, which highlights the need for continued adaptation of even the most efficacious interventions according to the characteristics of practice settings, and the broader context of program implementation (23).

The stakeholders suggested the use of technology to deliver the school mental health program training and collect outcomes data at-scale. This recommendation is in keeping with the rapid increase in availability of such technologies in low-income countries which can improve access to evidence-based mental health intervention in low resource settings (1). However, end-user involvement in the conception, design and prototyping of the technology platform was considered critical by the stakeholders for the success of such technological platform (24).

## Limitations

The use of ToC approach in the present study allowed us to examine expected outcomes from a diverse stakeholders’ perceptive while keeping in mind the existing limited resources. Although, the stakeholders identified parents and children as the key stakeholders for the success of school mental health program, we were only able to consult children and adolescents as part of needs assessment following the 1st ToC workshop which highlighted the need for such an intervention. Future ToC workshops will focus on involving children and adolescents to capture the outcomes of school mental health program that matter to them the most. Another limitation of our study was that stakeholders involved in the current study were from a limited geographical area of Pakistan (Rawalpindi district), hence, the generalizability of ToC map for the scale-up of school mental health program, developed on the views of these stakeholders, is limited to the population attending public schools in rural areas of Rawalpindi, Pakistan. The scale-up of SMHP in schools of urban area, especially in private educational institutions in Pakistan will require involvement of relevant stakeholders. Like any other consultative process, the possibility of social desirability bias in the responses of the stakeholders cannot be ruled out. However, since ToC map is a *‘hypothesized implementation pathway*’ and a working document, the next phase of implementation will verify the validity of proposed implementation plan based on the ToC map and will improve iteratively based on field experiences.

## Conclusion

The ToC approach was most suited to develop an implementation pathway for scaling-up school mental health program in public schools of rural Pakistan because it not only made the planning more rigorous and fruitful, but also allowed us to build on the local knowledge base of our stakeholders and end-users. It proved to be a powerful tool in promoting collaboration and involvement of diverse stakeholders and community members, increasing the chances of scaled-up implementation of school mental health program, encouraging collective change. Future workshops with adolescents will be valuable to bring end beneficiary perspective to scale-up of school based mental health programs in Pakistan.

## Data Availability

Not applicable.

## Abbreviations

ToC: Theory of Change
LMICs: Low or Middle-Income Countries
WHO: World Health Organization
SMHP: School Mental Health Program
EMRO: Eastern Mediterranean Region
SHINE: School Health Implementation Network, Eastern Mediterranean region
IoP-WCC: Institute of Psychiatry-World Health Organization Collaborating Centre for mental health research and training
UoL: University of Liverpool
HDRF: Human Development Research Foundation
AEOs: Assistant Education Officers
NIMH: National Institute of Mental Health

## Footnotes

1 https://www.nimh.nih.gov/about/organization/cgmhr/scaleuphubs/school-health-implementation-network-eastern-mediterranean-region-shine.shtml.
